# First principle study of ATiO$_3$ (A=Ti,Sr) materials for photovoltaic applications

**DOI:** 10.1007/s00894-023-05823-x

**Published:** 2024-01-10

**Authors:** Lynet Allan, Winfred M. Mulwa, R. E. Mapasha, Julius M. Mwabora, Robinson J. Musembi

**Affiliations:** 1 Department of Physics, Faculty of Science and Technology, University of Nairobi, P.O.Box 30197-00100, Nairobi, Kenya; 2 Department of Physics, Egerton University, P.O Box 536-20115, Egerton, Kenya; 3 Department of Physics, University of Pretoria, Private Bag x 20, Hatfield, Republic of South Africa

**Keywords:** Optical properties, Electronic and elastic properties, DFT + U, Spin orbit coupling, $\text {ATiO}_{3}$ (A=Ti, Sr)

## Abstract

**Context:**

The study investigates the impact of Hubbard *U* correction and spin-orbit coupling (SOC) on the structural, mechanical, electronic, and optical properties of $\text {Ti}_{2}\textrm{O}_{3}$ and $\text {SrTiO}_{3}$ compounds. The research is motivated by the potential applications of these materials in photovoltaics, with a focus on understanding their properties for such use. The ductility, ionicity, and mechanical stability of both compounds at zero pressure are assessed, indicating their potential as resilient materials. Also, the compounds display high refractive indices and absorption coefficients, indicating their suitability for solar harvesting applications. The predicted bandgaps align primarily with the UV–Vis areas of the electromagnetic spectrum, highlighting their potential in this domain.

**Methods:**

Computational techniques employed in this study are density functional theory (DFT) with and without spin-orbit coupling, as well as DFT+U methods, implemented using the Quantum ESPRESSO (QE) package. The study adopts the Perdew-Burke-Ernzerhof (PBE) exchange-correlation functional, while employing a plane-wave basis set with an energy cutoff of 50 Ry for wavefunctions and 500 Ry for charge density.

## Introduction

Due to the escalating global demand for energy and the adverse environmental impact of burning fossil fuels, the exploration of sustainable energy sources has become an imperative focus of research [1–3]. Over the past few decades, titanium-based oxides have garnered significant attention due to their potential applications in solar energy conversion and environmental remediation [3–5]. Titanium, a ubiquitous element on Earth, and its oxides, such as titanium dioxide ($\text {TiO}_{2}$) and $\text {ATiO}_{3}$ (A= Ba, Ca, Fe), have been the subject of intense investigation across various fields, including electronics, energy conversion, catalysis, and sensing [6–9].

Despite $\text {TiO}_{2}$’s wide band gap, it has been extensively studied for potential modifications to enhance solar harvesting capabilities [10, 11]. In contrast, reduced $\text {TiO}_{2}$ compounds like $\text {Ti}_{2}\textrm{O}_{3}$ have not received comparable attention. Yang Yang et al. previously discovered a cubic perovskite $\text {Ti}_{2}\textrm{O}_{3}$ unit cell at the $\text {Ti/SrTiO}_{3}$ interface, but the material’s properties were not thoroughly examined [11]. Our comprehensive research aims to determine the structural, electronic, elastic, and optical properties of $\text {Ti}_{2}\textrm{O}_{3}$ and $\text {SrTiO}_{3}$ for potential photovoltaic applications, building upon prior work [12].

The corundum structure of $\text {Ti}_{2}\textrm{O}_{3}$ has been established, with $\text {Ti}^{3+}$ atoms forming a mixture of edge, corner, and face-sharing $\text {TiO}_{6}$ octahedra bound to six equivalent $\textrm{O}^{-2}$ atoms [12]. Similarly, $\text {SrTiO}_{3}$ possesses a (cubic) perovskite structure, crystallizing in the tetragonal I4/mcm space group, where $\text {Sr}^{2+}$ is bound to 12 $\textrm{O}^{-2}$ atoms, creating $\text {SrO}_{(12)}$ cuboctahedra. Both materials’ stability and characteristics are investigated in this study to determine their suitability for solar harvesting applications.

The study further delves into titanium oxynitride compounds $\text {Ti}_{(n)}\textrm{N}_{(2)}\textrm{O}_{(2n-1)}$ with *n*=3, derived from pure $\text {Ti}_{2}\textrm{O}_{3}$, as recently examined by Allan et al. [13]. Despite exhibiting metallic qualities, $\text {Ti}_{2}\textrm{O}_{3}$ has been experimentally found to be insulating while also displaying semiconducting properties. Addressing the limitations of first-principles DFT, particularly its inability to accurately describe electronic band gaps in strongly correlated materials, we employ the Hubbard model (DFT+U) to more precisely estimate gaps and thoroughly analyze elastic and mechanical properties [2]. The investigation extends to the impact of spin-orbit coupling (SOC), an aspect often overlooked in experimental studies.

Considering the limited experimental studies on $\text {Ti}_{2}\textrm{O}_{3}$ and $\text {SrTiO}_{3}$ properties, this research aims to fill the gaps by utilizing the DFT approach with SOC and Hubbard *U*. The comprehensive examination covers structural, electronic, elastic, mechanical, and optical properties, paving the way for contemporary titanite-based technologies.

The article’s structure is organized as follows: Section 2 addresses the complexities essential for reproducing our computations in the future. Section 3 presents a thorough analysis of findings and discussions, with subsections covering the influence of SOC and Hubbard *U* on electronic properties (Section 3.2) and the structural properties of $\text {Ti}_{2}\textrm{O}_{3}$ and $\text {SrTiO}_{3}$ with and without SOC effects (Section 3.1). Elastic constants and mechanical properties are discussed in Section 3.3, while Section 3.4 explores the optical properties of $\text {Ti}_{2}\textrm{O}_{3}$ and $\text {SrTiO}_{3}$. Finally, Section 4 outlines the conclusions drawn from this comprehensive study.

## Computational details

The DFT, as implemented in the quantum espresso (QE) code by Giannozzi (2009), was used to calculate the structural, electronic, elastic, mechanical, and optical properties of the compounds $\text {Ti}_{2}\textrm{O}_{3}$ and $\text {SrTiO}_{3}$. The $\text {Ti}_{2}\textrm{O}_{3}$ and $\text {SrTiO}_{3}$ crystal structure input files were acquired from the materials project database [14], and the PWscf input files for DFT calculations were prepared using the materials cloud [15] input file generator implemented in QE. The projector augmented-wave function (PAW) approach, also known as PAW-PPS, was used to represent the electron-ion interaction. To define the exchange-correlation effect of electrons, the generalized gradient approximation (GGA) with the Perdew-Burke-Ernzerhof (PBE) [16] was used. The computations made use of the 50 Ry cut-off energy and 6$\times$6$\times$4 Monkhorst-Pack grid for the integration of the Brillouin zone. To determine the ground state structural properties, geometry optimization was done by calculating the total energy per unit cell at various lattice constant values. The elastic, electronic, and optical properties were computed using the optimized lattice constants. The effects of spin-orbit coupling (SOC) were taken into account while calculating the structural and electronic properties. Spin-orbit coupling (SOC) is an important relativistic effect that arises due to the interaction between the spin of an electron and its orbital motion around the nucleus. It is particularly relevant for heavy atoms, such as Sb and Bi, where the relativistic effects are stronger. In the context of electronic band structure calculations, SOC can have a significant impact on the energy levels and the associated wave functions. Specifically, SOC leads to a splitting of the degenerate energy levels associated with different angular momenta (l) and spin (s) quantum numbers. This splitting can be significant for some energy levels and can lead to the formation of new energy levels that were not present without SOC.

In the case of excitonic effects, SOC can play an important role in determining the probability of the formation of singlet and triplet excitons. Singlet excitons are formed by the excitation of an electron from the valence band to the conduction band, resulting in a bound electron–hole pair with opposite spin. In contrast, triplet excitons involve the excitation of an electron to a higher energy level with the same spin, leading to a bound electron–hole pair with parallel spins. Due to the SOC effect, the probability of generating triplet excitons is increased, as the SOC interaction leads to a coupling between the spin and orbital angular momentum, allowing for a mixing of the singlet and triplet states. This has important implications for the optical and electronic properties of materials, as the presence of triplet excitons can affect the efficiency of optoelectronic devices such as solar cells and light-emitting diodes. The Hubbard *U* correction was applied to the electronic structure calculations to address the well-known DFT underestimation problem while evaluating the effect of including *U* on the lattice parameter($\textrm{a}_{0}$) of the compounds.

## Results and discussions

### Structural properties

**Fig. 1:**
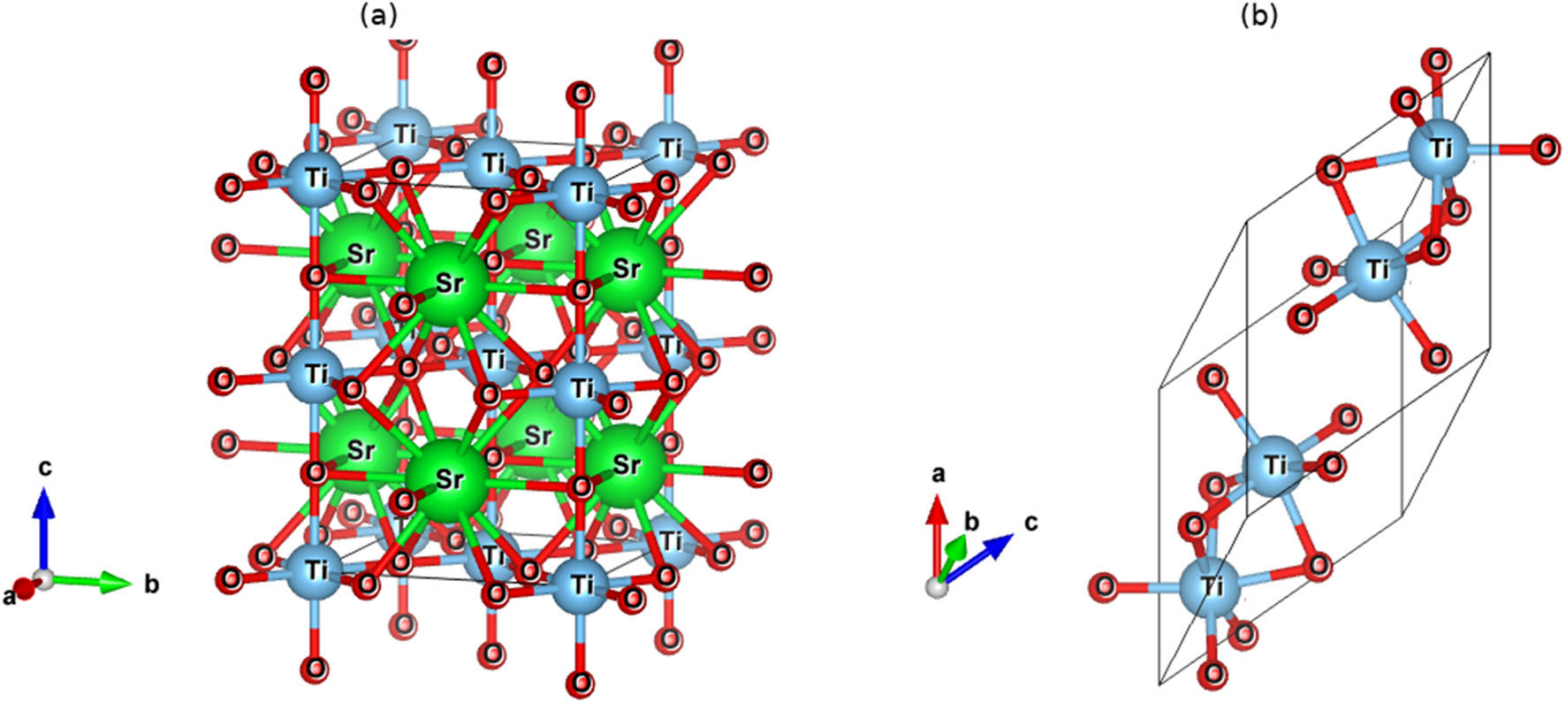
Optimized crystal structure for **a** $\text {SrTiO}_{3}$ and **b** $\text {Ti}_{2}\textrm{O}_{3}$ compounds

As per reports elsewhere [15], $\text {Ti}_{2}\textrm{O}_{3}$ and $\text {SrTiO}_{3}$ adopt a trigonal and tetragonal crystal system, respectively. The optimized crystal structures are presented in Fig. 1. One must determine the lattice parameter that minimizes the DFT total energy in order to arrive at the equilibrium structure. The bond strength for $\text {Ti}_{2}\textrm{O}_{3}$ and $\text {SrTiO}_{3}$ is shown in Table 1.

According to Table 1, the Ti-O bond in $\text {Ti}_{2}\textrm{O}_{3}$ is stronger than the Ti-Ti bond, but the Ti-O bond in $\text {SrTiO}_{3}$ is stronger than both the Ti-Sr and the Sr-O bonds. Because the volume per atom tends to rise with increasing atomic radius and so impacts the basis of the crystal lattice, the Sr-Sr bond is the weakest when the bond length is high. This difference is related to Mooser [17] chemical theory. A summary of the computed lattice parameters and other physical features can be seen in Table 2.

**Table 1:** Bond lengths for $\text {Ti}_{2}\textrm{O}_{3}$ and $\text {SrTiO}_{3}$

Material	Bond	Bond length
$\text {Ti}_{2}\textrm{O}_{3}$	Ti-O	2.04
	Ti-Ti	2.37
$\text {SrTiO}_{3}$	Sr-Ti	3.41
	Sr-O	2.78
	Sr-Sr	3.95

The equilibrium volume and energy of the ground states of the two compounds have some changes when the data were fitted to Murnaghan’s equation of state, as shown in Table 2.

### Effect of SOC and Hubbard *U* on electronic properties

**Fig. 2:**
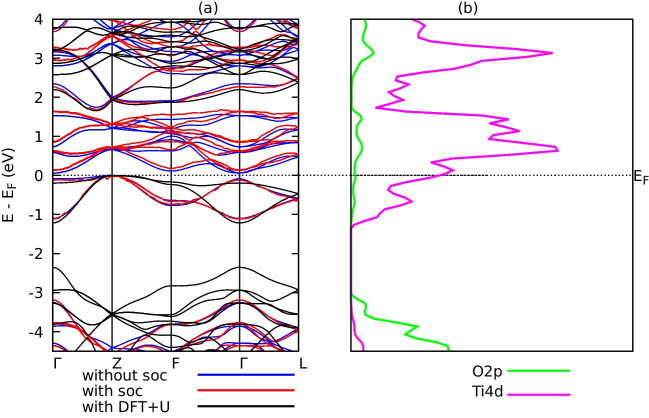
The calculated **a** band structures of $\text {Ti}_{2}\textrm{O}_{3}$ compounds without SOC effects (blue), with SOC (red), and with DFT+U (*U*=5 eV) (black). **b** The PDOS for $\text {Ti}_{2}\textrm{O}_{3}$ compound without SOC and without DFT+U effects

**Fig. 3:**
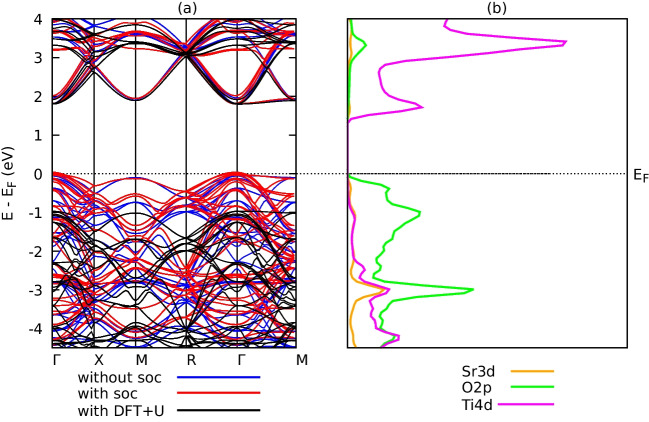
The calculated **a** band structures of $\text {SrTiO}_{3}$ compounds without SOC effects (blue), with SOC(red), and with DFT+U (*U*=5 eV) (black). **b** The PDOS for $\text {SrTiO}_{3}$ compound without SOC and without DFT+U effects

Figures 2 and 3 show the electronic band structures and projected density of states (PDOS) of the compounds $\text {Ti}_{2}\textrm{O}_{3}$ and $\text {SrTiO}_{3}$ computed using optimized crystal structures. Three calculations of the band structures were performed: one without SOC effects, one with the SOC effect, and one with Hubbard *U* corrections. For $\text {Ti}_{2}\textrm{O}_{3}$ and $\text {SrTiO}_{3}$, respectively, the choice of *U* values was based on past works on the same materials by references [13] and [18]. Hubbard *U*=5 eV and *U*=7 eV, respectively, were employed for band structure computations for $\text {Ti}_{2}\textrm{O}_{3}$ and $\text {SrTiO}_{3}$ since they had no apparent influence on the lattice constants.

**Table 2:** Effect of SOC on the structural properties of $\text {Ti}_{2}\textrm{O}_{3}$ and $\text {SrTiO}_{3}$

Structural properties	$\text {Ti}_{2}\textrm{O}_{3}$		$\text {SrTiO}_{3}$	
	Without SOC	With SOC	Without SOC	With SOC
Lattice Parameters $\textrm{a}_{0}$ (a.u)	9.933	10.404	10.535	10.849
Lattice parameter c/a	0.520	0.571	1.353	1.403
Bulk modulus $\textrm{B}_{o}$ (GPa)	166.4	137.0	217.1	218.2
Ground state energy	$-$673.7	$-$291.1	$-$1253.07	$-$1585.16
$$\textrm{E}_{o}$$ (Ry)				
Equilibrium volume	980.14	1126.09	1167.17	1277.00
$\textrm{V}_{o}$ (a.u)3				

**Table 3:** The calculated and experimental bandgaps

Materials	Calculated band gaps			Experimental gaps
	Without SOC	With SOC	With Hubbard *U*	
$\text {Ti}_{2}\textrm{O}_{3}$	0.0598	0.1311	1.667 (*U*=5eV)	1.67 [19]
$\text {SrTiO}_{3}$	1.612	1.7612	2.769 (*U*=7eV)	2.85 [20]

Narrow bandgaps have been reported for the compounds $\text {Ti}_{2}\textrm{O}_{3}$ and $\text {SrTiO}_{3}$. For example, $\text {Ti}_{2}\textrm{O}_{3}$ has electronic band gaps of 0.059 eV and 1.612 eV, respectively, without SOC, 0.131 eV, and 1.761 eV, respectively, with SOC, and 1.665 eV and 2.769 eV, respectively, with Hubbard *U* as shown in Figs. 2 and 3a. The summary of the calculated bandgaps is tabulated in Table 3.

**Table 4:** Computated elastic constants $\textrm{C}_{ij}$ (GPa) of $\text {Ti}_{2}\textrm{O}_{3}$ and $\text {SrTiO}_{3}$

Compound		$C_{11}$	$C_{12}$	$C_{13}$	$C_{14}$	$C_{33}$	$C_{44}$	$C_{66}$
$\text {Ti}_{2}\textrm{O}_{3}$	DFT	350.3	138.9	175.7	83.04	276.0	114.7	105.6
	DFT+U	285.3	102.1	113.4	3.073	228.4	121.5	91.6
SrTiO$_3$	DFT	338.3	110.6	105.7	-	351.6	116.5	122.3
	DFT+U	372.5	116.7	115.2	-	383.3	130.3	133.0

The band gaps were not significantly affected by the SOC according to computations of the band structure, however, Upon closer inspection, we found a slight change in band structure due to the presence of spin-orbit coupling (SOC). The SOC effect arises from the interaction between the electron’s spin and its motion. In our study, SOC leads to an energy splitting of the electronic bands, which in turn causes a slight expansion of the lattice. This expansion in the lattice leads to changes in the elastic properties of the materials. The DFT+U made better predictions of the band gaps in comparison to experimental values. The valence band maxima and minima for $\text {Ti}_{2}\textrm{O}3$ and $\text {SrTiO}_{3}$ occur at different symmetry positions in the Brillouin zone (Z-$\Gamma$) and (*Gamma*-M) for $\text {SrTiO}_{3}$, respectively, indicating that the two compounds are indirect band gap semiconductors. Calculations were made to determine the states that electrons might potentially occupy when projected onto atomic orbitals, or the projected density of states. However, only the outcomes of the PDOS without Hubbard *U* were considered because the effects of SOC and without Hubbard *U* did not significantly affect the types of dominating states in the PDOS as presented in Figs. 2b and 3b. The PDOS describes the orbital contributions to the formation of valence bands and conduction bands in the energy range from $-$4.5 to 4 eV. The orbitals O-2p and Ti-4d dominate the whole band structure, as seen in Fig. 2b, indicating that $\text {Ti}_{2}\textrm{O}_{3}$ shows semimetallic characteristics without Hubbard *U* effects. This is ascribed to the well-known DFT method’s underestimation of the electronic structure band gaps. In the $\text {Ti}_{2}\textrm{O}_{3}$ band structure depicted in Fig. 3, the O-2p states primarily form the bands in the energy area of $-$4.5 to $-$2.5 eV, whereas the Ti-4d states primarily form the bands in the energy region of $-$1.0 to 4 eV. The valence band of the $\text {SrTiO}_{3}$ compound as shown Fig. 3b is mostly generated by the O-2p.

**Table 5:** Mechanical properties of $\text {Ti}_{2}\textrm{O}_{3}$ and $\text {SrTiO}_{3}$ compounds

Compound		*B*	*E*	*G*	*B*/*G*	*n*
$\text {Ti}_{2}\textrm{O}_{3}$	DFT	217.0	250.2	95.7	2.26	0.31
	DFT+U	161.3	239.4	95.5	1.69	0.25
$\text {SrTiO}_{3}$	DFT	185.8	292.4	118.1	1.57	0.24
	DFT+U	202.5	322.9	130.8	1.54	0.23

### Mechanical and elastic properties

Six independent elastic constants are found in the trigonal and tetragonal crystal structures of $\text {Ti}_{2}\textrm{O}_{3}$ and $\text {SrTiO}_{3}$, respectively. $C_{11}$, $C_{12}$, $C_{13}$, $C_{14}$, $C_{33}$, and $C_{44}$ [21] are the independent elastic constants for trigonal $\text {Ti}_{2}\textrm{O}_{3}$. Equation (1) gives the required and sufficient conditions for trigonal $\text {Ti}_{2}\textrm{O}_{3}$ stability:

1 $$\begin{aligned} {\begin{matrix} C_{11}> \left| C_{12} \right| ;C_{44}> 0; \\ C_{13}^{2}< \frac{1}{2}C_{33}\left( C_{11} +C_{12}\right) ;\\ C_{14}^{2}< \frac{1}{2}C_{44}\left( C_{11} -C_{12}\right) = C_{44}C_{66};\\ C_{66} = \frac{1}{2}(C_{11}- C_{12}) \end{matrix}} \end{aligned}$$

The six independent elastic constants for tetragonal $\text {SrTiO}_{3}$ are $\textrm{C}_{11}$, $\textrm{C}_{12}$, $\textrm{C}_{13}$, $\textrm{C}_{14}$, $\textrm{C}_{33}$, $\textrm{C}_{44}$, and $\textrm{C}_{66}$ [21]. The following are the essential and sufficient requirements for the tetragonal crystal system’s elastic stability:

2 $$\begin{aligned} {\begin{matrix} C_{11}> \left| C_{12} \right| ;C_{44}> 0;\\ 2C_{13}^{2}< C_{33}\left( C_{11} + C_{12}\right) ; C_{66}> 0 \end{matrix}} \end{aligned}$$

The calculated elastic constants for both compounds are displayed in Table 4. According to the findings, both compounds are mechanically stable since they satisfy the stability requirements at the DFT and DFT + U levels. Table 5 lists the bulk modulus *B*, Young’s modulus *E*, shear modulus *G*, Pugh’s ratio *B* to *G*, and Poisson’s ratio *n*.

**Table 6:** Variations of the elastic moduli for $\text {Ti}_{2}\textrm{O}_{3}$ and $\text {SrTiO}_{3}$

	Young’s modulus		Linear compressibility		Shear modulus		Poisson’s ratio		Compound
	*E*min	*E*max	$\beta$min	$\beta$max	*G*min	*G*max	*n*min	*n*max	
Value	149.83 GPa	303.25 GPa	1.3689 $\text {TPa}^{-1}$	1.8795 $\text {TPa}^{-1}$	63.496 GPa	119.75 GPa	0.11271	0.56615	$\text {Ti}_{2}\textrm{O}_{3}$
Anisotropy	2.024		1.3730		1.886		5.0230		
Value	286.19 GPa	301.83 GPa	1.752 $\text {TPa}^{-1}$	1.8144 $\text {TPa}^{-1}$	113.87 GPa	122.31 GPa	0.22191	0.25707	$\text {SrTiO}_{3}$
Anisotropy	1.055		1.0357		1.074		1.1584		

From Table 6, $\text {Ti}_{2}\textrm{O}_{3}$ presents a broader range of Young’s modulus values, potentially offering more flexibility in tailoring the material’s stiffness to meet varying mechanical demands. Meanwhile, $\text {SrTiO}_{3}$, with its narrower range, may be advantageous if a consistent level of stiffness is desired throughout the application. The consideration of linear compressibility ($\beta$) is crucial, as $\text {Ti}_{2}\textrm{O}_{3}$’s higher compressibility may be advantageous in situations where controlled deformation is needed, while $\text {SrTiO}_{3}$’s lower compressibility can be a strength when stability under external pressure is paramount.

When it comes to shear resistance, both materials have similar ranges of shear modulus (*G*), with $\text {SrTiO}_{3}$ being slightly higher. The choice between them would depend on the specific requirements for shear resistance within the photovoltaic device. Poisson’s ratio (*n*) data indicate that $\text {Ti}_{2}\textrm{O}_{3}$ has a wider range of values, which can be valuable in applications where resistance to lateral deformation or the ability to control Poisson’s effect is critical. Depending on the particular application, either material may be preferred.

It is also important to consider anisotropy, where $\text {Ti}_{2}\textrm{O}_{3}$ displays higher values indicating significant directional variation in properties. Anisotropy can be advantageous for tailoring material behavior to different orientations within a device. However, it can also introduce complexities in design and analysis. In contrast, $\text {SrTiO}_{3}$ exhibits lower anisotropy, which may simplify material behavior considerations in certain applications.

**Table 7:** Eigenvalues of the stiffness matrix for $\text {Ti}_{2}\textrm{O}_{3}$ and $\text {SrTiO}_{3}$ in GPa

Compound	$\lambda _{1}$	$\lambda _{2}$	$\lambda _{3}$	$\lambda _{4}$	$\lambda _{5}$	$\lambda _{6}$
$\text {Ti}_{2}\textrm{O}_{3}$	100.66	112.24	113.29	119.75	212.81	653.07
$\text {SrTiO}_{3}$	116.56	116.56	122.31	227.75	243.02	557.65

**Fig. 4:**
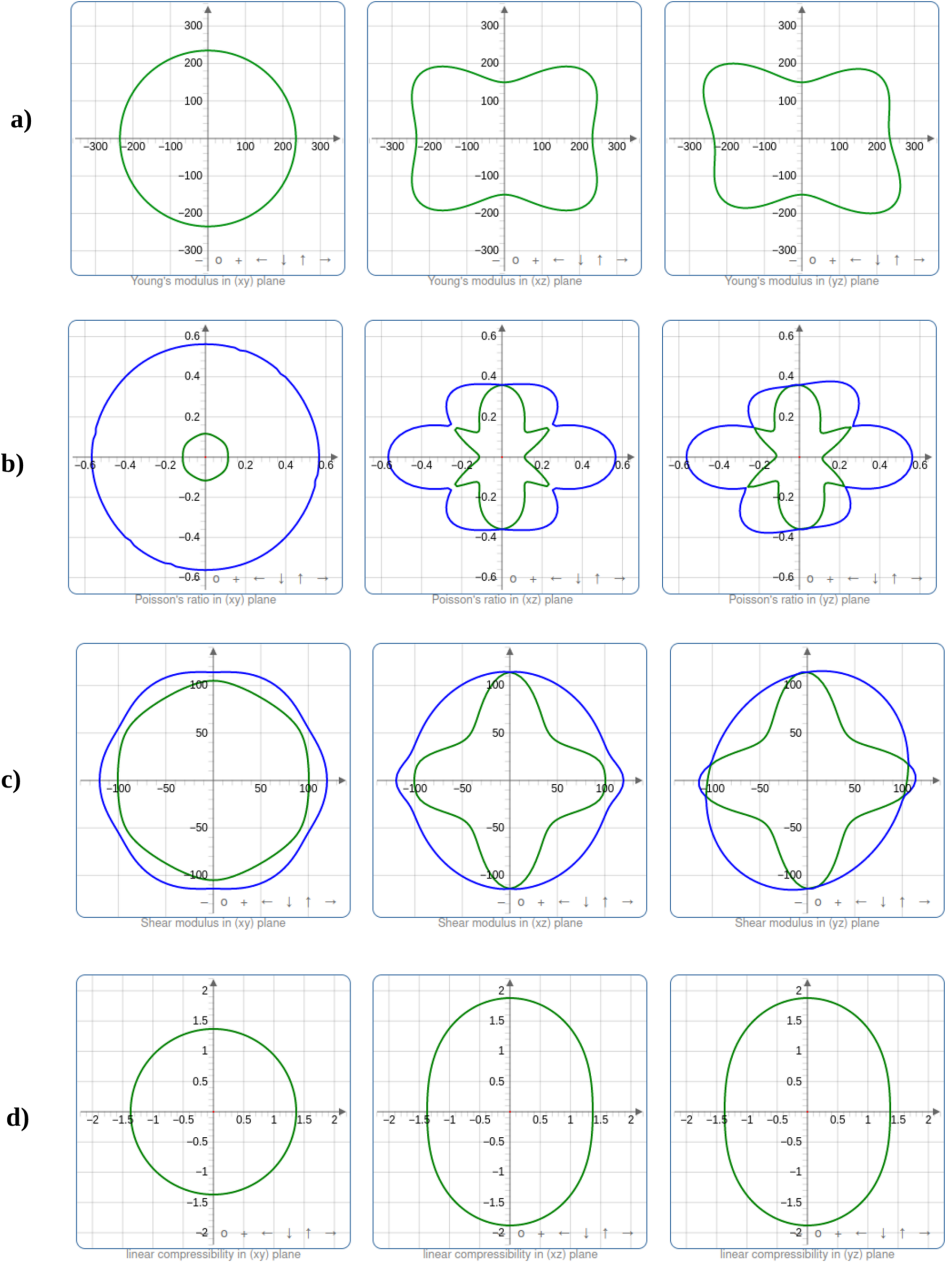
The spatial dependency of **a** Young’s modulus, **b** Poisson’s ratio, **c** shear modulus, and **d** linear compressibility for $\text {Ti}_{2}\textrm{O}_{3}$ (blue and green indicate the maximum and minimum values, respectively)

Table 7 presents eigenvalues, which represent the stiffness of the material along different crystallographic directions. Higher eigenvalues indicate greater stiffness in those specific directions. In the case of $\text {Ti}_{2}\textrm{O}_{3}$, eigenvalues are generally lower compared to $\text {SrTiO}_{3}$, indicating that $\text {Ti}_{2}\textrm{O}_{3}$ is less stiff in these directions. This suggests that $\text {Ti}_{2}\textrm{O}_{3}$ exhibits more anisotropic mechanical behavior as shown in Fig. 4.

Conversely, $\text {SrTiO}_{3}$ has higher eigenvalues, indicating higher stiffness in various crystallographic directions, suggesting that it is a stiffer material overall and exhibits more consistent mechanical behavior across different directions as can be observed in Fig. 4. The values of eigenvalues are crucial for understanding and predicting the mechanical behavior of materials, including their response to stress and strain in different orientations.

The bulk modulus (*B*) estimates the resistance to volume change brought on by applied external pressure. Since hard materials are predicted by large *B* values, $\text {Ti}_{2}\textrm{O}_{3}$ and $\text {SrTiO}_{3}$ are not hard materials according to computed bulk modulus values. With reference to the results obtained (in Table 2 when the lattice constants were fitted to the Murnaghan equation of state [22], it is evident that DFT+U provides a more accurate prediction of mechanical properties for $\text {Ti}_{2}\textrm{O}_{3}$. In addition, the size of the *B* is connected with the bond lengths of the crystal structures. The *B* value increases with shorter bond lengths [23]. The obtained bond lengths in $\text {Ti}_{2}\textrm{O}_{3}$ are shorter than those in the $\text {SrTiO}_{3}$ crystal structure based on the structural characteristics, leading to a larger value of *B* in the $\text {Ti}_{2}\textrm{O}_{3}$ compound as determined by DFT calculations. The Pugh ratio *B*/*G* and the Poisson ratio, *n*, respectively, govern the materials’ ductile (ionic) and brittle (covalent) properties. *B*/*G* < 1.75 is the limit for brittleness; otherwise, the material is referred to be ductile. The materials’ ductility and brittleness are also confirmed by the Cauchy pressure ($\textrm{C}_{12}$-$\textrm{C}_{44}$). In contrast, a negative value of the Cauchy pressure denotes brittleness and ductility, respectively. The critical value of the Poisson ratio is 0.26, and materials with *n* as 0.26 behave brittlely in all other circumstances. As a result, we can say that the compounds $\text {Ti}_{2}\textrm{O}_{3}$ and $\text {SrTiO}_{3}$ are ductile and strongly ionic in nature. Applying Young’s modulus value to a material yields its stiffness [23]. As a result, $\text {SrTiO}_{3}$ compound is stiffer than $\text {Ti}_{2}\textrm{O}_{3}$ because the greater the value of *E*, the stiffer the material.

**Fig. 5:**
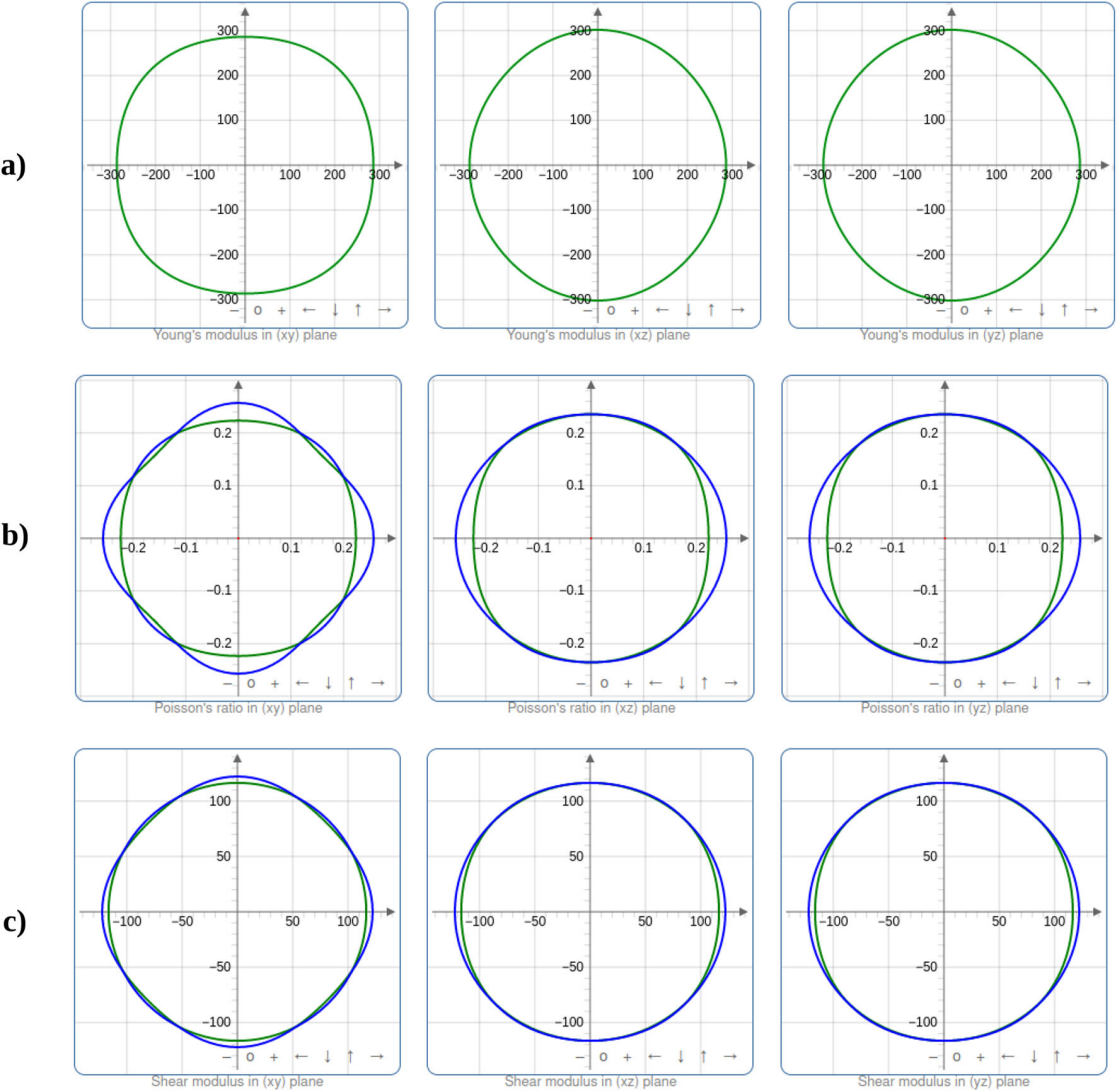
The spatial dependency of **a** Young’s modulus, **b** Poisson’s ratio, and **c** shear modulus, for $\text {SrTiO}_{3}$ (blue and green indicate the maximum and minimum values, respectively)

Figures 4 and 5 show the degree of anisotropy dependence on the deviation of a geometrical body from the spherical shape. In both cases, the representation is in a 2D shape. If a geometrical body is a spherical (3D) or circular (2D) shape, it exhibits isotropy, which is observed in *zy* the planes for Young’s modulus, shear modulus, Poisson’s ratio, and linear compressibility for $\text {Ti}_{2}\textrm{O}_{3}$, at slightly varying degrees, as shown in Fig. 4. However, the degree of anisotropy is observed to be asymmetric for Young’s modulus, shear modulus, Poisson’s ratio, and linear compressibility in *xz* and *yz* planes for $\text {Ti}_{2}\textrm{O}_{3}$. From Fig. 5, $\text {SrTiO}_{3}$ exhibits isotropy, which is observed in all the planes for Young’s modulus, shear modulus, and Poisson’s ratio at slightly varying degrees. The degree of anisotropy is observed to be symmetric for Young’s modulus, shear modulus, and Poisson’s ratio. The anisotropic variation for the calculated values of Young’s modulus, the shear modulus, and Poisson’s ratio are tabulated in Table 6 while the eigenvalues of the stiffness matrix for $\text {Ti}_{2}\textrm{O}_{3}$ and $\text {SrTiO}_{3}$ are displayed in Table 7.

### Optical properties

To understand how a solid interacts with light, it is very important to comprehend the optical properties of solids, which depend on their band gap. Calculating the dielectric function $\varepsilon \left( \omega \right)$, which describes the response of the electrons in the material to electromagnetic irradiation, is necessary to emphasize optical phenomena like reflection, transmission, and absorption. The dielectric function $\varepsilon \left( \omega \right)$ is given by the following:

3 $$\begin{aligned} \varepsilon \left( \omega \right) = \varepsilon _{1}\left( \omega \right) + i\varepsilon _{2}\left( \omega \right) \end{aligned}$$

**Fig. 6:**
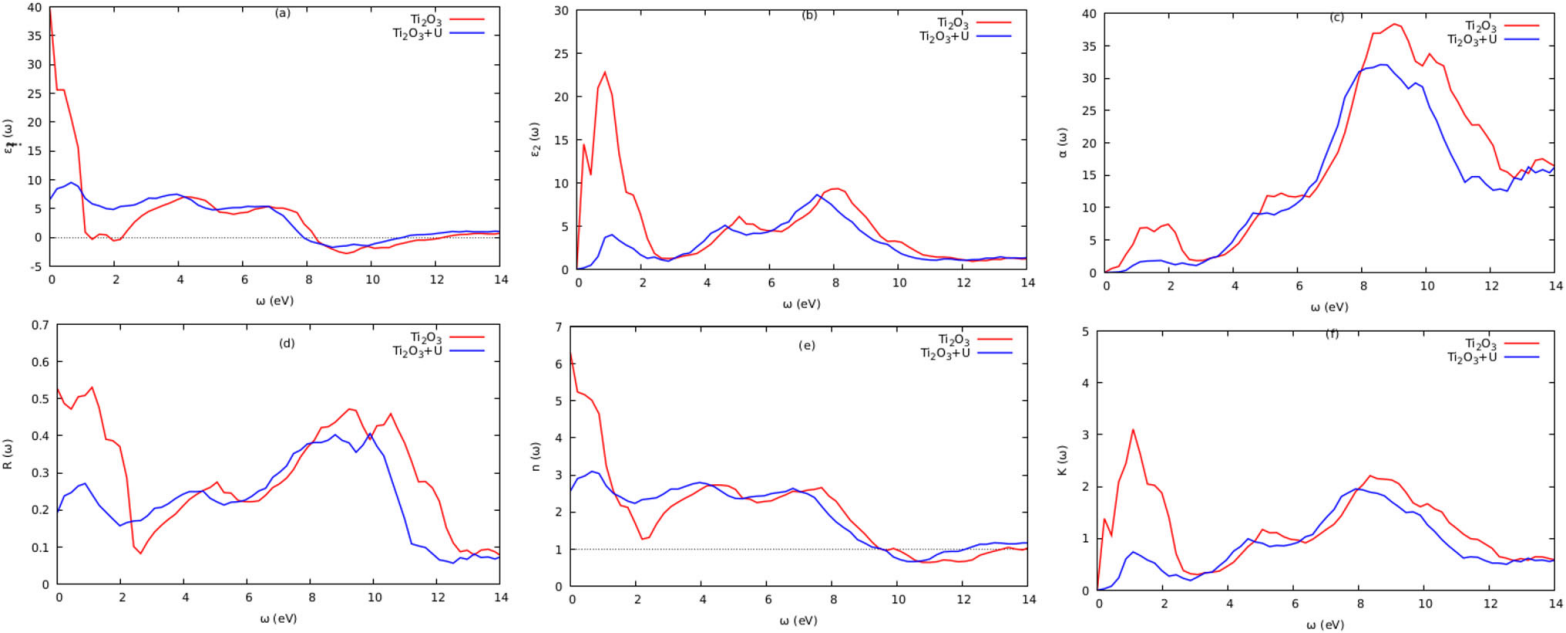
Optical properties for $\text {Ti}_{2}\textrm{O}_{3}$

**Fig. 7:**
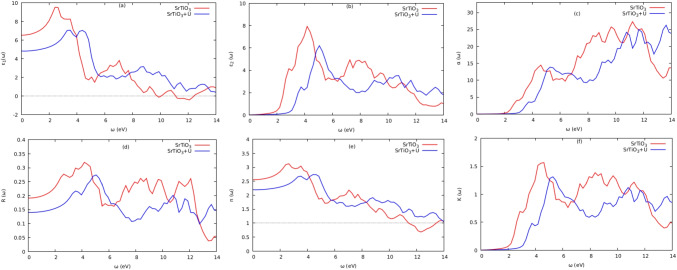
Optical properties for $\text {SrTiO}_{3}$

**Fig. 8:**
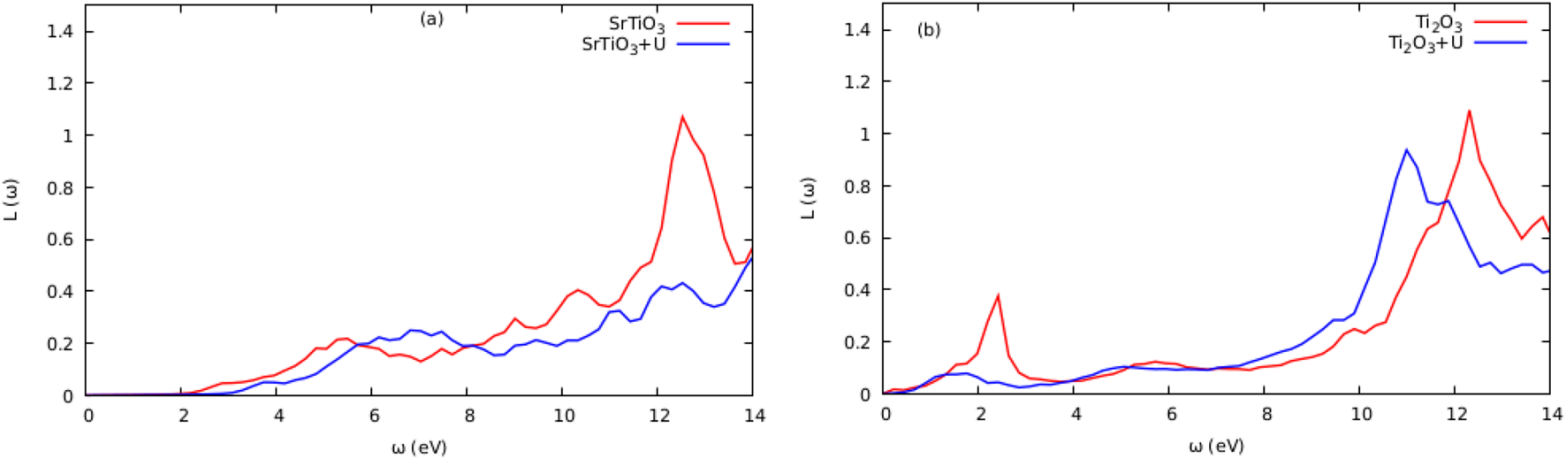
Energy loss $L\left( \omega \right)$ for $\text {ATiO}_{3}$

The real and imaginary components of the dielectric function are denoted by $\varepsilon _{1}\left( \omega \right)$ and $\varepsilon _{2}\left( \omega \right)$, respectively. The real part is connected to the material’s electronic polarizability, whereas the imagined part is connected to the material’s electronic absorption [24]. The real and imaginary components, respectively, explain how electromagnetic radiation disperses and absorbs through the appropriate material. According to the selection rules, the spectrum is determined by integrating the components of the aqueous dipole operator matrix between occupied and unoccupied wave functions over the Brillouin zone. This primarily relates to electronic structures and describes the material’s linear reaction to electromagnetic radiation. As a result, it controls how radiation behaves during propagation through a medium. The electron transition between the valence and conduction bands is represented by the imaginary component of the dielectric function. The other optical properties, such as reflectivity ($R\left( \omega \right)$), absorption ($\alpha \left( \omega \right)$), and refractivity ($n\left( \omega \right)$), can be obtained from the quantities ($\varepsilon _{1}\left( \omega \right)$) and ($\varepsilon _{2}\left( \omega \right)$). These are shown in Figs. 6, 7, and 8, respectively, and were calculated using the equations given below [4, 24, 25]:

4 $$\begin{aligned} \alpha \left( \omega \right) = \sqrt{2}\omega \left( \sqrt{\varepsilon _{1}^{2}\left( \omega \right) + \varepsilon _{2}^{2}\left( \omega \right) } - \varepsilon _{1}\left( \omega \right) \right) ^{1/2} \end{aligned}$$

5 $$\begin{aligned} {n\left( \omega \right) = \sqrt{2}\omega \left( \frac{\sqrt{\varepsilon _{1}^{2}\left( \omega \right) + \varepsilon _{2}^{2}\left( \omega \right) } - \varepsilon _{1}\left( \omega \right) }{2} \right) ^{1/2}} \end{aligned}$$

6 $$\begin{aligned} K\left( \omega \right) = \sqrt{2}\omega \left( \frac{\sqrt{\varepsilon _{1}^{2}\left( \omega \right) + \varepsilon _{2}^{2}\left( \omega \right) } - \varepsilon _{1}\left( \omega \right) }{2} \right) ^{1/2} \end{aligned}$$

7 $$\begin{aligned} L\left( \omega \right) = \frac{\varepsilon _{2}(\omega )}{\varepsilon _{1}^{2}\left( \omega \right) + \varepsilon _{2}^{2}(\omega )} \end{aligned}$$

8 $$\begin{aligned} R\left( \omega \right) = \frac{{(n - 1)}^{2} + K^{2}}{{(n + 1)}^{2} + K^{2}} \end{aligned}$$

Photon absorption in crystalline materials is described by the imaginary part ($\varepsilon _{2}\left( \omega \right)$) of the dielectric wave function [12]. The electronic transitions from the valence to the conduction bands are what cause the peaks in $\varepsilon _{2}\left( \omega \right)$ curves. The band gaps of the substances that fall within the visible region, which are <3.1 eV for $\text {Ti}_{2}\textrm{O}_{3}$ and $\text {SrTiO}_{3}$ compounds, are referenced by the absorption onsets in the $\alpha \left( \omega \right)$ curves. This implies that there are significant inter-band transitions. This makes the molecules $\text {Ti}_{2}\textrm{O}_{3}$ and $\text {SrTiO}_{3}$ attractive candidates for photovoltaic applications [4]. The static value, or $\varepsilon _{1}\left( \omega \right)$, sometimes known as the main characteristic of the $\varepsilon _{1}\left( \omega \right)$ curve at energy=0 [25]. This static value has a *n* ($\omega$) correlation with the material’s refractive index. The $\varepsilon _{1}\left( \omega \right)$ plot reached major peaks in low-energy regions, < 1.5eV and < 2.7eV for $\text {Ti}_{2}\textrm{O}_{3}$ and $\text {SrTiO}_{3}$, respectively. Until the $\varepsilon _{1}\left( \omega _{1}\right)$ values turned negative in the energy range of 8 to 12 eV, photon transmission persisted. The incident photon radiations are believed to be completely attenuated at this energy region [26], and the compounds exhibit metallic behavior. The computed refractive indices for $\text {SrTiO}_{3}$ at zero energies were 2.5 (with Hubbard *U*) and 2.3 (DFT$+$U) for the curves n ($\omega$), respectively. Within the visible region are the principal refractive index peaks for $\text {SrTiO}_{3}$ and $\text {Ti}_{2}\textrm{O}_{3}$ (with Hubbard *U*). In addition, it was discovered that the $\text {Ti}_{2}\textrm{O}_{3}$ and $\text {SrTiO}_{3}$ had substantial optical absorption in the 8–10 eV and 10–12 eV energy ranges, respectively. The UV–Vis spectrum is covered by the optical absorption coefficients of the compounds $\text {Ti}_{2}\textrm{O}_{3}$ and $\text {SrTiO}_{3}$. The computed absorption coefficients in the energy ranges of 2.0–13.5 eV encompassed a significant portion of the electromagnetic spectrum, demonstrating the suitability of these compounds for photovoltaic use. The reflectivity and the energy loss function, respectively, determine the surface behavior of the materials and the energy loss induced by rapid electrons entering a medium [25]. For $\text {Ti}_{2}\textrm{O}_{3}$ and $\text {SrTiO}_{3}$, the primary peaks of the reflectivity curves are seen in the regions 6–11 eV and 2–6 eV, respectively; the reflectivity declined beyond this region. Figure 8a shows the loss spectrum for both compounds, showing that there was no noticeable absorption in the visible ranges (b). Higher energy areas between 9.97 and 12.71 eV saw the main absorption peak. For $\text {SrTiO}_{3}$ and $\text {Ti}_{2}\textrm{O}_{3}$, respectively, the largest absorption peak occurred in locations with higher energies of >12 and >10 eV. For the materials $\text {SrTiO}_{3}$ and $\text {Ti}_{2}\textrm{O}_{3}$, the computed static dielectric constant $\varepsilon _{1}\left( 0\right)$, optical band gap $\Delta E_{OG}$, static refractive index *n* (0), static reflectivity *R* (0), and static loss function *L* (0) are tabulated in Table 8. The DFT + U technique enhanced the compounds’ optical characteristics, as seen in Table 8.

**Table 8:** Calculated static dielectric constant $\varepsilon _{1}\left( 0\right)$, optical band gap $\Delta E_{OG}$, static refractive index *n* (0), static reflectivity *R* (0), and static loss function *L* (0) for compounds $\text {SrTiO}_{3}$ and $\text {Ti}_{2}\textrm{O}_{3}$ compounds

Materials		$\varepsilon _{1}\left( 0\right)$	$\Delta E_{OG}$	*n* (0)	*R* (0)	*L* (0)
$\text {SrTiO}_{3}$	DFT	6.53	1.19	2.55	0.193	0.000355
	DFT+U	4.82	1.72	2.19	0.140	0.000239
Ti$_2$O$_3$	DFT	2.61	1.39	6.18	0.521	0.001520
	DFT+U	6.57	2.11	2.50	0.192	0.000230

## Conclusion

Using DFT (with and without SOC effects) and DFT+U methods implemented in the QE package, we have investigated the structural, electronic, elastic, mechanical, and optical properties of $\text {Ti}_{2}\textrm{O}_{3}$ and $\text {SrTiO}_{3}$ compounds. For the compound $\text {Ti}_{2}\textrm{O}_{3}$, equilibrium lattice constants of 9.93 a.u. without SOC and 10.40 a.u. with SOC were found, whereas for the compound $\text {SrTiO}_{3}$, equilibrium lattice constants of 10.53 a.u. and 10.84 a.u. without SOC and with SOC, respectively, were computed. Without SOC, $\text {Ti}_{2}\textrm{O}_{3}$ has an electronic band gap of 0.131 eV, whereas $\text {SrTiO}_{3}$ has an electronic band gap of 1.612 eV and 1.761 eV, respectively. The bandgaps for $\text {Ti}_{2}\textrm{O}_{3}$ and $\text {SrTiO}_{3}$ were calculated to be 1.665 eV and 2.769 eV, respectively, with the Hubbard *U* effects working in concert. These findings are in fair agreement with the published experimental findings. In both compounds, O-2p orbitals predominated in the valence bands whereas Ti-4d played a major role in the development of the conduction band. These properties were validated by DFT+U studies. $\text {Ti}_{2}\textrm{O}_{3}$ and $\text {SrTiO}_{3}$ were both found to be mechanically stable at zero pressure, ductile, and ionic, indicating their potential for use in resilient materials. DFT+U investigations also improved the optical characteristics of $\text {Ti}_{2}\textrm{O}_{3}$ and $\text {SrTiO}_{3}$.The predicted bandgaps, high refractive indices, high absorption coefficients, and broad energy coverage of the absorption coefficient spectra occurred primarily in the UV–Vis regions of the electromagnetic spectrum. According to this research, $\text {SrTiO}_{3}$ and $\text {Ti}_{2}\textrm{O}_{3}$ are excellent for photovoltaic applications.

## Data Availability

The authors will provide the source files and data upon request.
